# Communicating prognostic uncertainties in advanced multimorbidity: a multimethod qualitative study to co-design practice recommendations

**DOI:** 10.1007/s41999-025-01228-6

**Published:** 2025-05-24

**Authors:** Simon Noah Etkind, Rosanna Fennessy, James Wang, Rhian M. Simpson, Bernadette O’Neill, Stephen I. G. Barclay, Anna Spathis

**Affiliations:** 1 Primary Care Unit, Department of Public Health and Primary Care, University of Cambridge, East Forvie Building, Forvie Site, Addenbrookes Biomedical Campus, Robinson Way, Cambridge, CB20SZ UK; 2 Cambridge University Hospitals NHS Foundation Trust, Cambridge, UK; 3 Clinical School, University of Cambridge, Cambridge, UK; 4 Cambridgeshire and Peterborough NHS Foundation Trust, Cambridge, UK; 5 King’s College London, London, UK

**Keywords:** Uncertainty, Communication, Multimorbidity, Older adults, Co-design

## Abstract

**Purpose:**

Older adults with advanced multimorbidity face an uncertain future due to their unpredictable illness trajectory. Communication of such uncertainty is a core task in healthcare, but there is a limited evidence base to support communication of prognostic uncertainty in multimorbidity. We aimed to co-design clinical practice recommendations for communication of prognostic uncertainty in advanced multimorbidity, based on the lived experience and expertise of patients, carers, and healthcare professionals.

**Methods:**

Multi-method qualitative co-design study. In phase one, older adults with advanced multimorbidity, their informal carers, and health professionals participated in interviews and focus groups exploring experiences of uncertainty communication. Transcripts were analysed thematically. In phase two, attendees at two stakeholder events provided feedback on the findings and iteratively developed practice recommendations.

**Results:**

Fifteen older adults (median age 80, range 70–89), three informal carers, and 17 health professionals provided qualitative data. We then held workshops with 56 stakeholders. Participants considered that a personalised approach supported discussions of prognostic uncertainty; trusting care relationships, and regard for timing and preparation of all parties were needed. Participants agreed that discussions of uncertainty should proceed based on open and honest discussion within negotiated limits. Clinicians should signpost support and undertake parallel planning for the range of future possibilities to outline the boundaries of uncertainty.

**Conclusion:**

This study has developed recommendations for communication of prognostic uncertainty in older people with advanced multimorbidity, to support parallel planning for an unpredictable future. Implementing these recommendations, it may reduce negative impacts of uncertainty and improve care experience.

**Supplementary Information:**

The online version contains supplementary material available at 10.1007/s41999-025-01228-6.

## Key summary points

**Aim:**

To co-design clinical practice recommendations for communication of prognostic uncertainty in older people with advanced multimorbidity, based on the lived experience and expertise of patients, carers, and healthcare professionals.

**Findings:**

Discussions of uncertainty should proceed based on open and honest discussion within negotiated limits. Clinicians should signpost support and undertake parallel planning for the range of future possibilities to outline the boundaries of uncertainty.

**Message:**

These recommendations, iteratively developed and based on lived experience, can support clinicians to communicate the prognostic uncertainties which are prevalent and distressing in older adults with multiple serious illnesses.

**Supplementary Information:**

The online version contains supplementary material available at 10.1007/s41999-025-01228-6.

## Introduction

Uncertainty is pervasive in clinical practice: how it is appraised shapes its effect on wellbeing [1, 2]. In the context of serious illness, whilst some uncertainties can be resolved, for example, by seeking further information or undertaking additional investigations, many uncertainties commonly persist. If these irreducible uncertainties are appraised negatively, they can be distressing for patients and those caring for them, across multiple domains [3–6]. Uncertainty can also be a barrier to decision-making and care planning [7], with impacts on health professionals; if poorly tolerated it can result in moral distress [8], and higher risk of burnout [9, 10].

Irreducible uncertainty is particularly prevalent in advanced multimorbidity, which is increasingly common in older adults [11–14]. In situations of multimorbidity, complex and conflicting treatment pathways lacking an evidence base [15], fragmented care systems [16], and unpredictable illness trajectories[17] can combine to create many interrelated uncertainties, particularly regarding prognosis, which we define here in its broadest sense as the future course of illness, incorporating physical, psychological, and social domains of experience (see Table 1) [6, 18, 19].

**Table 1:** Definitions of concepts relevant to this research

Concept	Definition
Advanced multimorbidity	The co-existence of two or more serious or life-limiting illnesses [11–14]
Uncertainty	‘Known Unknowns’, characterised as inadequate understanding, a sense of incomplete, ambiguous or unreliable information, and conflicting alternatives [20]
Irreducible uncertainty	Uncertainty that cannot be fully eliminated or resolved. Also called aleatory uncertainty [21]. Prognostic uncertainty is a key example
Prognostic uncertainty	Uncertainty about the future course of illness, including survival, but incorporating broader physical, psychological, and social concerns such as future ability to undertake daily activities [19]

It may be possible to effectively address some uncertainties through communication to achieve reappraisal and a shared understanding [22]. If reappraised as ‘safe uncertainty’ [23], some of the distress from uncertainty may resolve and it can become an accepted part of illness [24], a protection against bad news [22], and for health professionals even an opportunity to learn and face intellectual challenges.[25]. Communicating uncertainty is therefore vital to facilitate wellbeing, decision-making, and effective care planning [26]. However, uncertainty communication can have both positive and negative effects on patient and carer distress and decision-making satisfaction, depending on how it is undertaken [18, 27–29].

The conversation analysis literature provides rich detail of what happens within an encounter; from this we know of helpful strategies to communicate aspects of prognostic uncertainty, particularly in the sense of likely length of survival [30, 31]. For example, it is helpful when the conversation is gently introduced through an indirect approach to assess readiness [32], using fishing questions to elicit patient concerns [32], or using hypothetical scenarios as a means to introduce uncertainty with more psychological safety [30, 32, 33]. Patients and carers often find it helpful to avoid spurious accuracy if clinicians provide best- and worst-case scenarios [34], or give general categorical estimates of prognosis ‘days’, ‘weeks’, and ‘months’ rather than specific numbers [35]. Clayton et al. synthesise some of these approaches in the PREPARED approach to prognostic communication [36], and Medendorp et al.’s scoping review provides an overarching framework for discussing uncertainty in general healthcare contexts [37].

However, evidence supporting these approaches is limited: communication of uncertainty in the context of multiple concurrent illnesses is underexplored, and we do not know what communication approaches meet the needs of people living with multimorbidity. Existing recommendations for uncertainty communication are not supported by empirical evidence from people with advanced multimorbidity [37]. Evidence-based approaches to addressing prognostic uncertainty in multimorbidity are needed, which incorporate patient, lay carer, and healthcare professional experience and expertise. We aimed to co-design clinical practice recommendations that address this knowledge gap.

## Methods

### Design

Multi-method qualitative co-design study, reported according to the GUIDED checklist (see supplementary information 1). In phase one, patient and carer interviews and health professional focus groups provided the base dataset. In phase two, stakeholders provided feedback on a summary of the qualitative findings to iteratively develop clinical practice recommendations. By involving a range stakeholders in the co-design process, we aimed to synthesise multiple viewpoints.

### Phase one: interviews and focus groups

#### Population and eligibility

Older adults with advanced multimorbidity, those providing informal care to this group (i.e., family caregivers), and health professionals supporting this population.

We operationalised this as follows:

- Patients: Age ≥ 65, with ≥ 2 serious or life-limiting illnesses (diagnoses included in the Murtagh et al. estimate of conditions conferring palliative care need, modified to include frailty, and musculoskeletal conditions limiting physical function, which are independent predictors of poor outcome) (see supplementary information 2)
- Carers: an informal (family or friend) caregiver identified by patients who met the above criteria.
- Health professionals: Professionals involved in the care of patients meeting the above criteria and who have a role in addressing prognostic uncertainty.

We excluded patients with physical or cognitive impairment, such that they would be unable to participate in an interview, those living beyond the catchment areas of study sites, and those unable to complete an interview in English (if an interpreter could not be arranged).

We sought a purposive sample, based on maximum-variation of patient diagnoses, age range, and health professional roles. We did not aim to include a comprehensive sample of all combinations of multimorbidity. We estimated, based on the focused, in-depth nature of the information we wished to collect, that 12–18 patients plus identified carers, and 15–25 health professionals would provide sufficient information power to generate evidence on recommendations for communication which could be expanded on and refined in the stakeholder workshops. We conducted analysis alongside data collection to ascertain when we had collected sufficient data.

#### Setting, participant identification, and recruitment

Clinical staff identified patients at palliative care ‘living well’ services, and bed-based rehabilitation services. Carers were identified by consenting patients. We chose these settings on the basis that patients receiving such services were more likely to have serious or life-limiting diagnoses. In the UK patients attending palliative care, ‘living well’ services usually have a prognosis of months to years. Health professionals from community palliative care, geriatrics, and rehabilitation services were identified through existing contact networks within participating organisations; All provided written consent.

#### Focus groups and interviews

Participants provided demographic information and took part in an in-depth interview (patient and carers) or focus group (health professionals). Data collection was face-to-face, except for one focus group, which was held online. Patients and carers were interviewed separately unless joint interviews were requested. We used topic guides that explored experiences of prognostic uncertainty; experiences of uncertainty communication; preferences and views for such communication (see Supplementary information 3). Interviews and focus groups were audio-recorded, transcribed verbatim, and anonymised prior to analysis.

#### Analysis

Following data familiarisation, transcripts were analysed with inductive thematic analysis. Initially, analysis of patient and carer interviews was conducted separately from HCP focus group transcripts. RF (female research psychologist) and SE (male palliative care clinician) coded the transcripts for areas relevant to uncertainty communication, considering both statements of preferred communication approaches, and inferring preferred approaches from experiences of poor communication. Three interviews and one focus group transcript were double-coded; differences were discussed and resolved by RF and SE. We grouped codes into descriptive themes and then considered latent concepts as we generated analytic themes. The research team then met to discuss and compare patient, carer, and HCP findings, identify differences and similarities, and build an overarching framework of draft recommendations. At this stage, we considered existing models of uncertainty communication [6, 37], and used the categories of Medendorp’s model (preparing for the discussion, informing patients, and helping patients deal with uncertainty) to structure the draft recommendations.

### Phase two: stakeholder consultation

We held stakeholder workshops where the qualitative findings were presented and discussed. These workshops were held sequentially with 3 months between them to enable iterative development of the recommendations.

#### Population and eligibility

The population and eligibility for both phases of the study were the same. Qualitative study participants were subsequently invited to attend stakeholder consultations, and we invited additional health professionals, patients, and carers with relevant experience as we envisaged some participants would be unable to contribute to both phases.

#### Data collection

Workshops involved presentation of the qualitative findings, followed by group discussion. After providing written consent, participants provided general feedback on the qualitative findings, focusing particularly on missing areas or changes needed. To refine the recommendations, we then asked participants to discuss and comment on their relevance in example case scenarios (see supplementary information 4). We produced these scenarios with Patient and Public Involvement (PPI) members to highlight challenges for communicating prognostic uncertainty (e.g., variation in awareness of patients and clinicians of the illness situation, reluctance to raise the issue, and unrealistic expectations). The scenarios were closely based on PPI members’ lived experiences. The stakeholder workshops were not recorded to allow for open conversation across multiple groups; data collection took the form of copious notes and researcher reflections.

#### Analysis

After each workshop, we read and discussed the notes, synthesising the issues raised. We compared stakeholder views to the earlier qualitative findings and identified areas of synergy and difference, adjusting the recommendations accordingly.

### Patient and public involvement

A group of patients, carers, and members of the public met regularly throughout the study. Crucially, PPI members provided feedback on the findings and shared their lived experiences of situations of uncertainty. These experiences formed the core of the case studies for the stakeholder workshops. PPI members also advised on study documents, including the topic guide.

## Results

### Participant details

In the qualitative phase, 15 patients and three carers provided interviews, and 17 health professionals took part in three focus groups (six doctors, five nurses and healthcare assistants, four physiotherapists one occupational therapist, one chaplain). The most frequent patient diagnoses were heart failure, chronic lung disease, and advanced cancer. All patient participants were pre-frail or frail **(see **Tables 2–3**).** All patient-carer dyads opted to be interviewed jointly.

**Table 2:** Qualitative study patient and carer details

Characteristic	Measurement	Patients	Carers
Age^1^	Median (range)	81 (70–89)	76 (65–77)
Gender^1^	Female	8 female	2 Female
	Male	7 male	1 Male
Ethnicity^1^		White 15	White 3
Number of eligible diagnoses^2^	Median (range)	3 (2–4)	
Number of participants with each eligible diagnosis^2,3^	Cancer	7	
	Cardiovascular disease	11	
	Respiratory disease	10	
	Renal disease	2	
	Liver disease	1	
	Dementia/neurological disease	3	
	Musculoskeletal disease	8	
Clinical frailty score [38]	Median (range)	5 (4–6)	

1 self-reported

2 diagnoses included in modified Murtagh et al.’s estimate, see supplementary information

3 Each participant had at least two eligible diagnoses

**Table 3:** Qualitative study healthcare professional details

	Number	Age (median, range)^1^	Gender^1^	Ethnicity^1^	Years of experience in role^1^
Focus group 1:	6	51 (34–66)	4 Female 2 Male	Asian 1 Mixed 1 White 4	1–4 years: 1 10 + years: 5
Focus group 2:	7	44 (20–55)	6 Female 1 Male	Asian 4 White 3	< 1 year: 1 5–9 years: 1 10 + years: 5
Focus Group 3:	4	37 (30–42)	2 Female 2 Male	White 4	5–9 years: 3 10 + years: 1

1. self-reported

The first stakeholder workshop was attended by a mixed group of 48 health professionals, researchers, and patient/carer representatives. To ensure patient and carer voices were adequately represented, we held a second workshop with 8 patients and carers **(See **Table 4).

**Table 4:** Stakeholder participants

Stakeholder workshop 1 (*n* = 48)^1^	
Patient representative/carer	8
Doctor (GP, palliative care, geriatrician)	21
Nurse (community nurse, palliative care nurse)	13
Paramedic	1
Social worker	2
Pharmacist	1
Researcher	6
Clinical management/leadership role	2
Other	1

Stakeholder workshop 2 (*n* = 8)	
Patients	6^2^
Carers	2^3^

1. Participants could select multiple roles. 2. Four of these also provided qualitative interviews. 3. One of these also provided a qualitative interview

### Underpinning approaches to communicate uncertainty

From the interview and focus group data, two principles underpinned optimal approaches to communicating prognostic uncertainty in advanced multimorbidity: the need for a personalised approach, and a trusting care relationship.

#### Personalised approach

Whilst person-centredness is core to all care in serious illness, participants considered a personalised approach was particularly important in advanced multimorbidity, because every person’s illness situation was characterised by a unique set of prognostic uncertainties.

I think about the future and what's going to happen, when you're first diagnosed I do wish they wouldn't, they said the prognosis was three to five years, and they tell everybody that … and I wish they wouldn't because everybody's different.

Male with three qualifying diagnoses

Patients and carers expressed a variety of responses to prognostic uncertainty—some tended to accept it, whereas others found the idea of an uncertain future more distressing. Health professionals recognised this range of responses and felt that these would affect their approach to communication.

Maybe about how kind of emotionally resilient we feel they are, and how much you feel they can cope with more or less uncertainty, yeah, something that kind of like not knowing, some people are kind of personalities they want deadlines, they want dates and times, so I suppose it’s trying to know your patient to know how you can tailor what you say to their needs, and their ability to cope.

Health care professional – rehabilitation setting

#### Trusting care relationship

Participants considered discussions about prognostic uncertainty to be sensitive; most highlighted the importance of continuity and an ongoing care relationship to maintain trust when broaching the topic. Without such a relationship, expressions of health professional uncertainty could be interpreted as signs of incompetence rather than honesty. Given the multiple illness trajectories, it was preferable for a care provider well versed with the overall situation to be involved in addressing prognostic uncertainty, rather than a series of specialists familiar with only one aspect. A good care relationship enabled openness about prognostic uncertainty whilst maintaining trust.

the ideal would be that you have a warm and sort of supportive relationship and then you’re able to provide that kind of comfort of saying, you know “I’m willing to put myself on the line and say I don’t know”, which maybe is better overall.

Health care professional – geriatrics setting

### Communication of uncertainty in practice

We generated three themes in relation to optimal prognostic uncertainty communication; a) timing and preparation, b) communicating honestly and openly within negotiated limits, and c) signposting support and planning for future possibilities.

#### Timing and preparation

The timing of conversations about prognostic uncertainty was recognised to be delicate. Health professionals were particularly aware of this, and considered potential triggers for uncertainty communication, which could be system related, e.g., at transitions of care; patient related, e.g., a new diagnosis, change in plan for care, change in health status; or due to a specific concern raised by patients or carers. Prognostic uncertainty was often increased by inconsistent information between different specialists and there was a tension between seeking clarification from specialists, and attempting to address uncertainty in a timely fashion.

they’re under maybe several different specialisms, they may be under, you know, renal, they may have some oncology, they may have some, you know, urology things going on, and they find it that the coordination of their care very uncertain … they may need a prostate procedure but actually they’ve got cardiology things going on and they’re waiting to hear from cardiology and it’s that uncertainty “am I gonna get this procedure I need because I need, you know, maybe two other specialities to agree it before it happens?”

Health care professional, palliative care setting

Carers noted the importance of their presence at such discussions to support a patient. This helped to support recall, understanding, and decision-making in uncertain situations.

Uh huh. And then they were talking about oh, do you want to be resuscitated and things like that and that just come, well out of the blue, especially for my husband because like I said to you, he’s not aware of medical terms or anything like that and I, not translate but I tell him how he’d understand it like, what it actually means because they say things that you don’t know the terminology.

Carer of multimorbid patient

Thorough HCP preparation for conversations about prognostic uncertainty was considered important, though participants did not think that waiting for the optimal time or for all information should be an excuse to put off discussions. HCPs identified the challenge in determining whether prognostic uncertainties were due to a personal lack of knowledge or were true unknowns, and to prepare by attempting to gather available information in advance. Finally, adequate training was considered essential to ensure that health professionals are sufficiently familiar with future trajectories in multimorbidity and are skilled to hold sensitive discussions.

I think that we get comfortable in our own little zones and dealing with the uncertainties that we’re familiar with, but if we take ourselves out of that zone then we might struggle again.

Health care professional, palliative care setting

#### Communicate openly within negotiated limits

When communicating prognostic uncertainty, the clear consensus view was that openness and honesty are essential to enable a full understanding of the situation. Further, openness was linked to a trusting relationship in that many participants were concerned about the idea of health professionals withholding information. Sharing uncertainty openly was generally well received.

if you were to turn around and say to me today, well, you've got lung cancer and you've got an aneurysm that … might kill you tomorrow, the lung cancer might drag on a bit and that'll mean you can't breathe and go to oxygen and all that, I'm quite, I'm quite happy with all that because that's what's going to happen.

Male with four qualifying diagnoses

Patient and carer participants mainly recognised that the future course of multimorbidity was inherently unpredictable, and considered that even if the honest answer was ‘nobody knows’, being overt about prognostic uncertainty could turn an ‘unknown unknown’, into a more manageable ‘known unknown’. Achieving a shared understanding in this way could therefore be reassuring.

well, I think, if you know what’s happening and what’s going to happen, well reasonably what’s going to happen, I think you cope better. And if you don’t know, you’re always worried what’s next, or what should I do.

Female with two qualifying diagnoses

However, prognostic uncertainty was often not communicated openly; some participants experienced reluctance from health professionals to provide information. Health professionals themselves acknowledged that being completely honest about uncertainty, particularly one’s own uncertainty, required a degree of courage. Challenges in discussing uncertainty openly depended on the type of uncertainty; for example, it was considered less acceptable to share personal uncertainties, than to share uncertainties due to the lack of an evidence base in for treatments in multimorbidity, or due to the unpredictable illness trajectory.

I think it’s probably because people expect us to know the answer don’t they? So I think what you don’t want to convey to a patient is that it’s a lack of your own knowledge or your own ability as to why you can’t make a diagnosis and I think that sometimes is the challenge isn’t it? It’s communicating it in a way that still makes them feel like they’ve got confidence and trust in you to look after their relative or to look after them whilst understanding that you don’t have all the answers and that’s a difficult balance sometimes to tread.

Healthcare professional—geriatrics setting

Participants expressed nuanced views as to the limits of openness, and communication preferences depended on the individual and their response to prognostic uncertainty. Whereas many participants did express a desire for complete openness, others acknowledged the potential harms of knowing about uncertainties. Therefore, a degree of negotiation as to information preferences was usually deemed appropriate.

I don’t know whether I would or not really... I don’t know, I think I’d like to know then I think I’d… I don’t know. [laughs] You can think of it a lot of ways, can’t you? … I mean, I think if somebody did tell me I would worry about it but I wouldn’t be like my husband. My husband would really be frantic about it, you know…”

Female patient with three qualifying diagnoses

Participants raised circumstances where discussing prognostic uncertainty openly was unhelpful, for example where acknowledging uncertainty might lead to more patient or carer distress, or if patients did not wish to know what the future might hold. This was particularly true where a large number of uncertainties were recognised by health professionals in a given situation—communicating all of these together could become overwhelming. HCPs felt conflicted between the desire to avoid paternalism, and recognition that in some circumstances, it might be appropriate to hold prognostic uncertainty themselves and not overtly discuss it.

There are some people for whom they absolutely don’t want to touch on these topics at all and for them what is good care is just not addressing the uncertainty… if they had to think about it then that brings all of the demons out into the open, if they can just pretend like these uncertainties don’t exist then they’ll be happier.

Health care professional, palliative care setting

Some participants suggested that use of existing approaches to communication might help those with less experience to raise prognostic uncertainty, e.g., by prompting about possible future scenarios in situations of advanced multimorbidity. It was considered that these should be adapted to individual situations. One patient participant suggested a question prompt list could be useful.

I don’t know what questions to ask, the obvious ones, you know, but if, yeah, if you had some sort of, well I was going to say crib sheet, I don’t know if that’s the right word and you thought, well I can ask him that and if you can pick and choose the questions you want to ask I think it’s helpful.

Male with three qualifying diagnoses

#### Signpost support and plan for future possibilities

It was important to participants that communication of prognostic uncertainty should not stop at discussion of its existence. Doing so could leave patients and carers feeling lost and facing future unknowns without support. A vital second part of the conversation is, therefore, offering support and making a plan for future eventualities. Supportive statements were considered helpful regardless of whether uncertainty could be resolved or not.

I don't mind them saying they're not sure. I do mind them saying we don't know, goodbye. If they say they're not sure, but we'll have a look at this, fine, you know? Please, do.

Female with three qualifying diagnoses

They may still have questions about, you know, the trajectory of their illness or, you know, especially the neurological conditions. Will I lose power in my legs? Will I be able to feed my…? They’ll still have all of those questions but they may have, you know, a bit of reassurance that there is different services out there that can support.

Health care professional, palliative care setting

A helpful plan was one which addressed a range of future possibilities rather than a single most likely scenario. Doing so could ‘map’ the boundaries of uncertainty, without committing to false certainties about the future. Planning for optimistic scenarios to maintain hope, whilst discussing realistic worst-case scenarios in a parallel planning approach was considered useful, particularly when the future illness trajectory was unpredictable. One participant used this approach to support confidence with discharge planning.

I do a lot of discharge planning, get patients home, and as I’ve said uncertainty of going home, if they’re going to be safe and stuff, so we work towards setting them up as a worst-case scenario, because a lot of the patients are worried of going home so they need that extra reassurance, more equipment going in, just to make sure that the transition is safe for when they do get discharged from the ward

Health care professional – rehabilitation setting

Avoiding spurious certainty in future plans was felt to be important. Nevertheless, where possible to end the conversation on a concrete and practical note, this was considered useful.

But at the same time you actually bring them back to [the] present actually, you take them [to the] future, give them information, empower them and then bring back to [the] present time, what actually I can do currently for you.

Health care professional, palliative care setting

Finally, communication of prognostic uncertainty needed to be translatable across clinical contexts given the fragmented nature of multimorbidity care. Documentation of such conversations was, therefore, particularly important, and could avoid multiple distressing conversations.

Document her views almost verbatim and share it so she’s not continually interrogated.

Notes from stakeholder workshop

### Refining the recommendations using patient scenarios

In the stakeholder workshops, participants were presented with the qualitative findings, and discussed the themes in the context of example case studies (see supplementary information) to refine draft recommendations. Participants focused on the three themes relating to communicating uncertainty in practice as described above.

#### Timing and preparation

Participants discussed the importance of setting up conversations about prognostic uncertainty well, and considered who might be best placed to hold such discussions. They highlighted the necessity of ensuring good prior knowledge of the overall health situation and status of all morbidities in advance. Stakeholders reflected on the timing of these conversation and there was consensus that in a scenario where recovery was uncertain, a conversation should be held as soon as uncertainty of recovery was identified as a possibility. One scenario involved a patient who was reluctant to discuss the future; in this situation, participants first considered whether a conversation about prognostic uncertainty was needed. Most participants recognised that the patient’s reluctance to discuss it could be an important coping mechanism for her, so finding an opportunity or trigger to open the discussion was vital. All agreed that the timing of such conversations could not necessarily be controlled and there was an opportunistic element.

Basic data—she’s likely to die within the year. Difficult to initiate if she’s well, easier if unwell.

Notes from stakeholder workshop

#### Communicating openly within negotiated limits

It was recognised that starting a conversation with the specific agenda of discussing uncertainty might be jarring, and participants considered how to introduce the topic of prognostic uncertainty in a non-threatening manner. One suggestion was to follow the patient’s preferences and values, framing any discussion of uncertainty around these and the illness context, for example using the phrase “What can we put in place to let you live day-by-day?”. Stakeholders also noted that there might be difference in what family members want to know compared to patients, and felt that health professionals should consider potential variation between patient and family information needs when thinking about communicating. In response to concerns about providing too much information, e.g., about all possibilities relating to multiple illness trajectories, participants highlighted the need to negotiate information needs. One group suggested that ‘drip feeding’ information would be a helpful approach:

Gauge understanding, and desire for information. “Drip-feed” uncertainty by sharing “I’m worried that … could be a sign of”. Careful with wording – e.g. “improved” vs “cured”.

Notes from stakeholder workshop

#### Signposting support and planning for future possibilities

Participants agreed that planning for future possibilities was an important part of conversations about prognostic uncertainty in multimorbidity, and highlighted the importance of parallel planning approaches. The AMBER care bundle, an intervention used in situations of uncertain recovery which includes daily review of the plan, was considered a potentially useful intervention.

Parallel planning—promote hope, while discussing range of possibilities.

AMBER care bundle used in cases of uncertain outcome—review plan daily and communicate directly with pt.

Notes from stakeholder workshop

**Fig. 1:**
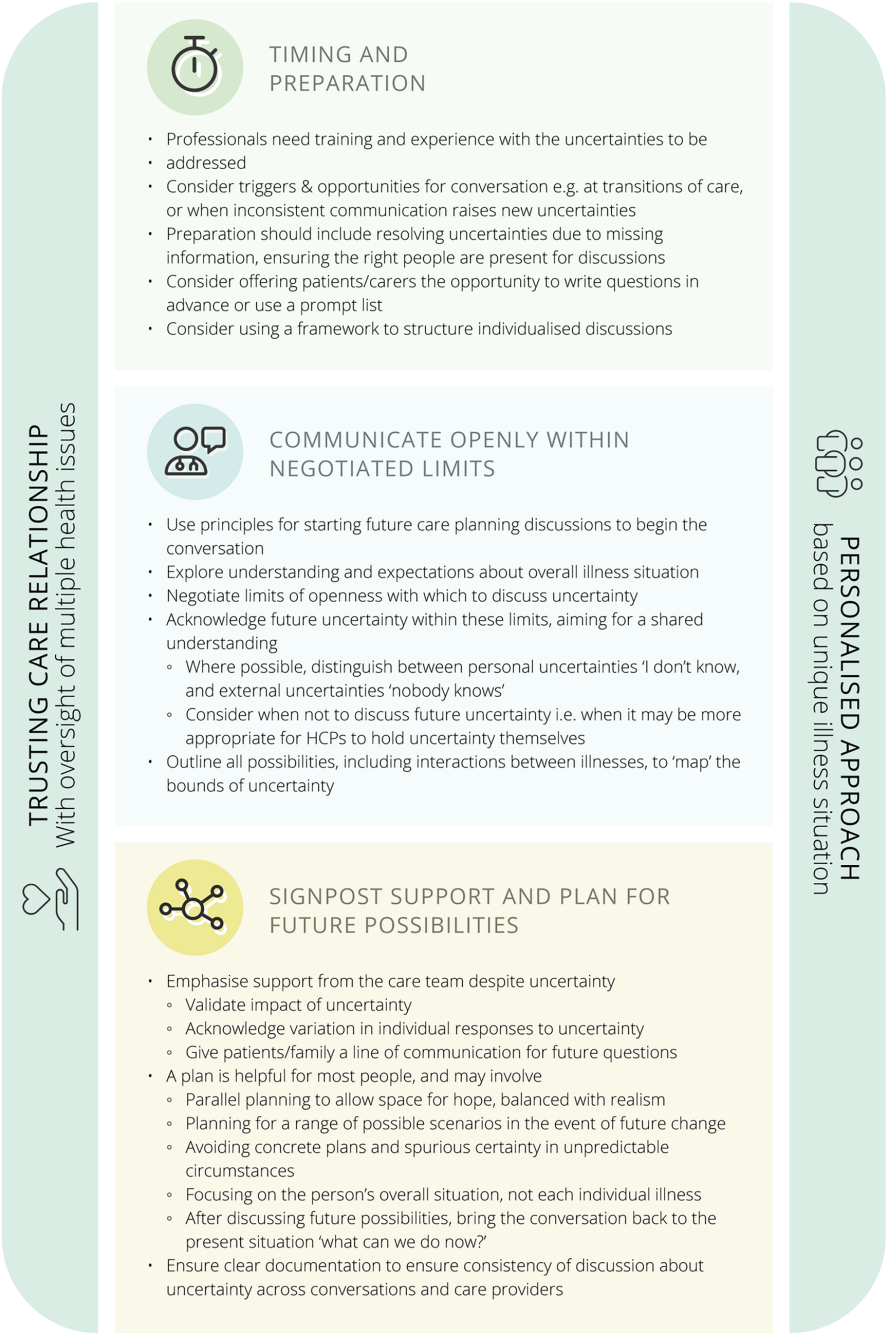
Recommendations to communicate prognostic uncertainty in multimorbidity

We developed the following recommendations for communication of prognostic uncertainty grounded in the data from the qualitative phase, and refined by stakeholders at the workshops. See Fig. 1.

## Discussion and conclusion

This study has developed recommendations for communication of prognostic uncertainty in advanced multimorbidity, underpinned by iterative in-depth qualitative research. Participants identified an overarching need for a personalised approach, and felt that uncertainty should be addressed in the context of a trusting care relationship, with due regard for timing and preparation of all parties. Discussions of uncertainty should proceed based on open and honest discussion within negotiated limits, signposting support and planning for future possibilities. These recommendations do not specify a single optimal approach to uncertainty here, recognising rather that a range of techniques may be helpful depending on the individual situation.

We describe a communication approach grounded in the context of advanced multimorbidity. Since the concept of making a plan is central to almost all healthcare encounters, these recommendations inevitably share similarities with more general approaches to communicating uncertainty [37], and communicating about the future [36]. What is different here is the acknowledgement that in the context of multiple concurrent illness trajectories, a single plan is rarely enough. In particular, participants repeatedly highlighted that planning should take in the wide range of possibilities inherent to the multimorbidity illness trajectory. Making parallel plans for possible future scenarios focuses on patient experiences rather than on statistical probabilities of dying, which may support generation of more helpful narratives about care. There is some evidence that a parallel planning approach is helpful in paediatric contexts, usually in relation to single illnesses with uncertain outcome, but parallel planning is not widely used in multimorbidity care [39]. Taken together, outlining and planning for uncertain future scenarios may offer a way to ‘map out’ the terrain of an uncertain future in multimorbidity, identifying the bounds of uncertainty and to some degree safely containing it.

Participants’ statements of challenges in being open about uncertainty resonate with models of uncertainty communication that highlight the importance of acknowledging uncertainty [40]. However, there was a minority view that uncertainty should not always be shared. To identify when and to what degree uncertainty should be communicated can be as difficult a challenge as actually communicating it sensitively. The idea of negotiated openness presented here aligns with work identifying degree of involvement in care as an element of person-centredness [41], and evidence that a minority of people do not wish to talk about prognosis at all [42].

### Practice implications

Conversations relevant to these recommendations are increasingly common as the prevalence of multimorbidity rises [13], and we have endeavoured to co-design an approach that can easily be implemented clinically. A clear finding of this study was that health professionals need preparation and experience to discuss prognostic uncertainty in this context, and that timing is crucial. However, consensus recommendations regarding broader conversations in palliative and end-of-life care emphasise that “the priority should be to have the conversation, rather than to wait for an ideal time to have it” [43]. The unpredictable nature of multimorbidity makes it more challenging to identify the ‘right’ time for prognostic discussions in this population; but this challenge should be used as a trigger to discussing prognosis rather than an excuse not to do so. Participants here noted the converse, and they reported reluctance from the point of view of health professionals to discuss uncertainty, despite the general preference of patients and carers for openness. Poor health professional tolerance of multilayered uncertainty may inhibit prognostic discussions [44]. It is, therefore, particularly important that tolerance of uncertainty, and the ability to initiate prognostic conversations in the absence of certainty is developed during HCP training [45].

All participants in this study were in some form experts in addressing and communicating uncertainty in multimorbidity, either by training or lived experience. These findings, therefore, represent a distillation of expert experience. We do not yet know whether synthesising expert approaches to communication in this way will support those with less extensive experience to communicate about prognostic uncertainty [46]. Future work is needed to implement and evaluate the recommendations developed here and consider adapting them to other clinical scenarios.

### Strengths and limitations

We conducted extensive, multi-perspective primary qualitative research, incorporating lived experience at every stage, including in the example scenarios, and we were thus able to use stakeholder experiences as source data to develop and refine the recommendations. A wide range of HCPs were represented. The two rounds of stakeholder consultation enabled iterative refining of the recommendations and repeated sense-checks to ensure a clinically relevant final product. PPI provided continual oversight to ensure relevance.

We were only able to recruit three carers for qualitative interviews, as most patient participants did not identify a carer. This means that the carer perspective is underrepresented. However, several carers did participate in the stakeholder workshops, and we specifically held an additional separate workshop with patients and carers to enable them to express their views. Stakeholder feedback suggested that the recommendations did resonate with carer experience. The patient and carer sample did not reflect the ethnic diversity of local populations, so our findings may not be transferrable to these populations. It is important for future work to specifically seek out diverse views and experiences of uncertainty communication, particularly to explore a range of cultural perspectives on the limits of openness. We did not recruit any General Practitioners to the health professional component, so their views were not incorporated—a future study should explore their views on this topic. Health professionals who attended the stakeholder consultations likely had a pre-existing interest in uncertainty, so views of those not already engaged in this topic may not be represented; however, our goal was to gather expert views. Future work should explore a wider range of professional views on uncertainty communication.

## Conclusion

Communicating about the uncertain future course of illness in the growing number of people with advanced multimorbidity can potentially improve care experience and planning. Participants in this study felt that prognostic uncertainty can be helpfully addressed through parallel planning for possible future scenarios, if the degree of openness is negotiated first. These recommendations build on the existing communications frameworks, by offering an approach to communicate about prognostic uncertainty specific to advanced multimorbidity. The next step is to implement and evaluate them.

## Supplementary Information

Below is the link to the electronic supplementary material.

Supplementary file1 (DOCX 34 KB)

## Data Availability

Due to the potentially sensitive nature of the topics discussed, our ethical approval limits how we can share the data. Whilst the qualitative data that this article is based on cannot be shared publicly, the anonymised data can be made available on request by email to the corresponding author, or to the department data manager: pcu_data@medschl.cam.ac.uk.

## Declarations

### Prior presentations

Initial data from this study were presented at the European Association for Palliative Care Congress 2024.
