# Supplementary material for: Communicating prognostic uncertainties in advanced multimorbidity: a multimethod qualitative study to co-design practice recommendations
Source: Eur Geriatr Med. 2025 May 24;16(4):1217–29. doi: 10.1007/s41999-025-01228-6 (PMC12378655; doi:10.1007/s41999-025-01228-6)
Supplement: Supplementary file 1 — Supplementary file1 (DOCX 34 KB) [file 41999_2025_1228_MOESM1_ESM.docx]

**Supplementary information**

**Supplementary information 1. GUIDED Checklist**

| **Item description** | **Explanation** | **Page in manuscript where item is located** |
| --- | --- | --- |
| 1. Report the context for which the intervention was developed. | Understanding the context in which an intervention was developed informs readers about the suitability and transferability of the intervention to the context in which they are considering evaluating, adapting or using the intervention. Context here can include place, organisational and wider sociopolitical factors that may influence the development and/or delivery of the intervention (15). | background |
| 2. Report the purpose of the intervention development process. | Clearly describing the purpose of the intervention specifies what it sets out to achieve. The purpose may be informed by research priorities, for example those identified in systematic reviews, evidence gaps set out in practice guidance such as The National Institute for Health and Care Excellence or specific prioritisation exercises such as those undertaken with patients and practitioners through the James Lind Alliance. | aim |
| 3. Report the target population for the intervention development process. | The target population is the population that will potentially benefit from the intervention – this may include patients, clinicians, and/or members of the public. If the target population is clearly described then readers will be able to understand the relevance of the intervention to their own research or practice. Health inequalities, gender and ethnicity are features of the target population that may be relevant to intervention development processes. | Background and methods |
| 4. Report how any published intervention development approach contributed to the development process | Many formal intervention development approaches exist and are used to guide the intervention development process (e.g. 6Squid (16) or The Person Based Approach to Intervention Development (17)). Where a formal intervention development approach is used, it is helpful to describe the process that was followed, including any deviations. More general approaches to intervention development also exist and have been categorised as follows (3):- Target Population-centred intervention development; evidence and theory-based intervention development; partnership intervention development; implementation-based intervention development; efficacybased intervention development; step or phased-based intervention development; and intervention-specific intervention development (3). These approaches do not always have specific guidance that describe their use. Nevertheless, it is helpful to give a rich description of how any published approach was operationalised | methods |
| 5. Report how evidence from different sources informed the intervention development process. | Intervention development is often based on published evidence and/or primary data that has been collected to inform the intervention development process. It is useful to describe and reference all forms of evidence and data that have informed the development of the intervention because evidence bases can change rapidly, and to explain the manner in which the evidence and/or data was used. Understanding what evidence was and was not available at the time of intervention development can help readers to assess transferability to their current situation. | Methods |
| 6. Report how/if published theory informed intervention development. | Reporting whether and how theory informed the intervention development process aids the reader’s understanding of the theoretical rationale that underpins the intervention. Though not mentioned in the e-Delphi or consensus meeting, it became increasingly apparent through the development of our guidance that this theory item could relate to either existing published theory or programme theory | Methods - analysis |
| 7. Report any use of components from an existing intervention in the current intervention development process. | Some interventions are developed with components that have been adopted from existing interventions. Clearly identifying components that have been adopted or adapted and acknowledging their original source helps the reader to understand and distinguish between the novel and adopted components of the new intervention. | Methods - analysis |
| 8. Report any guiding principles, people or factors that were prioritised when making decisions during the intervention development process. | Reporting any guiding principles that governed the development of the application helps the reader to understand the authors’ reasoning behind the decisions that were made. These could include the examples of particular populations who views are being considered when designing the intervention, the modality that is viewed as being most appropriate, design features considered important for the target population, or the potential for the intervention to be scaled up. | Methods |

| **Item description Explanation** | | **Page in manuscript where item is located** |
| --- | --- | --- |
| Report how stakeholders contributed to the intervention development process. | Potential stakeholders can include patient and community representatives, local and national policy makers, health care providers and those paying for or commissioning health care. Each of these groups may influence the intervention development process in different ways. Specifying how differing groups of stakeholders contributed to the intervention development process helps the reader to understand how stakeholders were involved and the degree of influence they had on the overall process. Further detail on how to integrate stakeholder contributions within intervention reporting are available(19). | Methods, results |
| 10. Report how the intervention changed in content and format from the start of the intervention development process. | Intervention development is frequently an iterative process. The conclusion of the initial phase of intervention development does not necessarily mean that all uncertainties have been addressed. It is helpful to list remaining uncertainties such as the intervention intensity, mode of delivery, materials, procedures, or type of location that the intervention is most suitable for. This can guide other researchers to potential future areas of research and practitioners about uncertainties relevant to their healthcare context. | ?discussion |
| 11. Report any changes to interventions required or likely to be required for subgroups. | Specifying any changes that the intervention development team perceive are required for the intervention to be delivered or tailored to specific subgroups enables readers to understand the applicability of the intervention to their target population or context. These changes could include changes to personnel delivering the intervention, to the content of the intervention, or to the mode of delivery of the intervention. | To be considered in future research |
| 12. Report important uncertainties at the end of the intervention development process. | Intervention development is frequently an iterative process. The conclusion of the initial phase of intervention development does not necessarily mean that all uncertainties have been addressed. It is helpful to list remaining uncertainties such as the intervention intensity, mode of delivery, materials, procedures, or type of location that the intervention is most suitable for. This can guide other researchers to potential future areas of research and practitioners about uncertainties relevant to their healthcare context. | discussion |
| 13. Follow TIDieR guidance when describing the developed intervention. | Interventions have been poorly reported for a number of years. In response to this, internationally recognized guidance has been published to support the high quality reporting of health care? interventions^5^and public health interventions^14^. This guidance should therefore be followed when describing a developed intervention. | This has been followed where possible, however as this is the initial phase of intervention development, not all information is yet available. |
| 14. Report the intervention development process in an open access format. | Unless reports of intervention development are available people considering using an intervention cannot understand the process that was undertaken and make a judgement about its appropriateness to their context. It also limits cumulative learning about intervention development methodology and observed consequences at later evaluation, translation and implementation stages. Reporting intervention development in an open access (Gold or Green) publishing format increases the accessibility and visibility of intervention development research and makes it more likely to be read and used. Potential platforms for open access publication of intervention development include open access journal publications, freely accessible funder reports or a study web-page that details the intervention development process. | Open access publication will be sought |

**Supplementary information 2. Conditions included in assessment of advanced multimorbidity**

**Table 1: ICD 10 codes used to assess presence of advanced multimorbidity.** (Based on the Murtagh minimal estimate of palliative care need) [1]

| Grouping | Code | Conditions included |
| --- | --- | --- |
| Cancer | C00 – C97 | All malignant neoplasms^1^ |
| Organ Failure | I00 – I52 (excl. I12 &I13)  J40-47, J96  I12, I13, N17, N18, N28  K70 - 77 | Symptomatic heart disease and heart failure^2^  Chronic lower respiratory disease, respiratory failure  Reno-vascular disease, renal failure with CKD 4/5.^3^  Symptomatic Liver disease^4^ |
| Dementia | F01, F03, G30, R54 | Dementia, Vascular dementia, Alzheimer’s disease, Senility.^5^ |
| Other | G10, G12.2, G20, G23.1, G35, G90.3  I60 – I69 | Huntington’s disease, Motor neurone disease, Parkinson’s disease, Progressive supranuclear palsy, Multiple sclerosis, Multi system atrophy.  Haemorrhagic, ischaemic and unspecified stroke.  Long term musculoskeletal conditions such as osteoarthritis, osteoporosis if limiting daily activity.  Any other serious life limiting illness not included above, including clinical syndromes such as frailty. |

1. Exclude non metastatic skin cancers and cancer being treated with curative intent
2. Exclude if: hypertension only, atrial fibrillation only, myocardial infarction with full recovery, NYHA class 1 (asymptomatic) heart failure
3. Exclude if CKD 1 – 3
4. Include cirrhosis of any cause, non-alcoholic fatty liver disease
5. Only if retain capacity to participate

1. Murtagh FE, Bausewein C, Verne J, Groeneveld EI, Kaloki YE, Higginson IJ. How many people need palliative care? A study developing and comparing methods for population-based estimates. Palliative medicine. 2014;28(1):49-58.

**Supplementary information 3. Topic guides**

**(a) HCP topic guide**

**Study title: Experiences and communication of future uncertainty in multimorbidity:**

**an intervention co-design study**

**Topic guide for healthcare professional focus groups.**

Section 1: Experience of uncertainty

**Main question.** **To start with I’d like to ask each of you to introduce yourself and describe your experience of managing future unknowns when caring for older people with multimorbidity**

*Sub questions and prompts.*

- Think of an older patient you have looked after who had multiple advanced illnesses. When caring for that patient, or others like them, what uncertainties were prominent?
- What effects do you think uncertainty has on your patients and their carers?
- What effect does uncertainty have on you and your practice? What challenges does it bring?

Section 2: Response to uncertainty:

**Main question. When you recognise future uncertainty, how do you respond?**

*Sub questions and prompts.*

- How do you respond when you recognise future uncertainty?
  - Do you always share uncertainty?
- How do you respond when your patient/carer raises uncertainty?
- What responses to uncertainty do you think are most helpful/harmful?

Section 3: Communicating uncertainty

**Main question. What approaches do you take when communicating future uncertainty in multimorbidity?**

*Sub questions and prompts.*

- How can future uncertainty best be communicated?
  - What works well in such conversations? What are your top tips?
  - What steps do you take within the conversation? How do you structure the conversation?
  - What are facilitators or barriers to holding such conversations?
- Do you tailor the conversation to patients, and if so how?
  - If patients accept/avoid/address uncertainty, how does this change your communication?
- How does the presence of multimorbidity affect conversations about future uncertainty?
- What would change how you communicate about uncertainty or the information you share?
- What would a toolkit to aid communication of uncertainty look like?

**(b) Patient and carer topic guide**

**Study title: Experiences and communication of future uncertainty in multimorbidity:**

**an intervention co-design study**

**Topic guide for patient and carer interviews**

Section 1: Experience of uncertainty and uncertainty communication

**Question.** **To start with I’d like to ask [each of] you to tell me about your daily lives, how they are affected by illness, and how you feel about the future**

*Sub questions and prompts [ask patient and carer in turn].*

- What are you uncertain about when you think ahead to the future? Can you give an example?
- What effect does uncertainty have on you?
- Have you had conversations about uncertainty with health professionals? Please tell me about them.
- [If not expressing uncertainty about the future] how have you overcome uncertainties previously?

Section 2: Response to uncertainty

**Question. When you feel uncertain about the future, how do you respond?**

*Sub questions and prompts.*

- When they feel uncertain about their future some people try to avoid discussing it, whereas others wish to discuss their future in detail, is either of these true for you? Could you say more?
- To what extent do you prefer to know what might happen in future with your illness?
- If health professionals are uncertain about your future illness, what would you want them to do?
- [if not uncertain about the future] how would you respond if you did feel uncertain?

Section 3: Communicating uncertainty

**Question. What is the best way to discuss uncertainty about your future with health professionals?**

*Sub questions and prompts.*

- Who should raise uncertainty first? What would help you to ask about it if you wished to?
- What affects how easy or difficult it is to discuss uncertainty?
- How much information should be shared? What affects this?
- What words or phrases are helpful when discussing uncertainty?
- What might a toolkit to support uncertainty communication look like?
- Is there anything else that would improve conversations about uncertainty?

**Supplementary information 4. Case scenarios for stakeholder workshops**

Scenario 1.

Alice is a 78 year old with heart failure and lung disease (Chronic Obstructive Pulmonary Disease, or COPD). She has had several heart attacks, and has had a number of procedures including a heart bypass operation in the last few years. She has also had several hospital admissions with chest infections related to her COPD. She is currently at home where she has carers to support her twice a day.

Her health is very unpredictable on a day-to-day basis. How do you think this uncertainty should be talked about if she:

1. Wanted to know as much as possible about everything that could happen?
2. Said she wanted to live day by day and did not want to discuss the future?
3. Declined to discuss uncertainty saying that ‘I’ve always recovered before, and I will again’?

Scenario 2

James is an 86 year old who has been managing well at home. He was recently diagnosed with colon cancer and was offered an operation, though was informed of higher than usual risks due to his medical history of reduced kidney function.

The operation was successful, and the surgical team believe all the cancer was removed. However, 3 weeks after the operation, he has not improved as hoped and remains unwell in hospital. He is still unable to get out of bed unaided and his future progress is uncertain.

How would you approach a discussion about future care if

1. You are the patient/family member and don’t know why things are not improving or what to expect?
2. You are a healthcare professional and still expect improvement. You aren’t sure why things are not improving?
3. You are a healthcare professional and recognise recovery is uncertain?
